# Microbial occurrence in liquid nitrogen storage tanks: a challenge for cryobanking?

**DOI:** 10.1007/s00253-021-11531-4

**Published:** 2021-09-24

**Authors:** Felizitas Bajerski, Manuela Nagel, Joerg Overmann

**Affiliations:** 1 Leibniz Institute DSMZ-German Collection of Microorganisms and Cell Cultures, Inhoffenstraße 7B, 38124 Brunswick, Germany; 2 Genebank Department, Leibniz Institute of Plant Genetics and Crop Plant Research (IPK), 06466 Seeland OT Gatersleben, Germany; 3 Institute of Microbiology, Braunschweig University of Technology, 38106 Brunswick, Germany

**Keywords:** Bioarchive, Biobanking, Biorepository, Microbial contamination, Cryobank, Cryoconservation, Cryopreservation, Risk/quality management, Safe storage

## Abstract

**Abstract:**

Modern biobanks maintain valuable living materials for medical diagnostics, reproduction medicine, and conservation purposes. To guarantee high quality during long-term storage and to avoid metabolic activities, cryostorage is often conducted in the N_2_ vapour phase or in liquid nitrogen (LN) at temperatures below − 150 °C. One potential risk of cryostorage is microbial cross contamination in the LN storage tanks. The current review summarises data on the occurrence of microorganisms that may compromise the safety and quality of biological materials during long-term storage. We assess the potential for the microbial contamination of LN in storage tanks holding different biological materials based on the detection by culture-based and molecular approaches. The samples themselves, the LN, the human microbiome, and the surrounding environment are possible routes of contamination and can cause cross contaminations via the LN phase. In general, the results showed that LN is typically not the source of major contaminations and only a few studies provided evidence for a risk of microbial cross contamination. So far, culture-based and culture-independent techniques detected only low amounts of microbial cells, indicating that cross contamination may occur at a very low frequency. To further minimise the potential risk of microbial cross contaminations, we recommend reducing the formation of ice crystals in cryotanks that can entrap environmental microorganisms and using sealed or second sample packing. A short survey demonstrated the awareness for microbial contaminations of storage containers among different culture collections. Although most participants consider the risk of cross contaminations in LN storage tanks as low, they prevent potential contaminations by using sealed devices and − 150 °C freezers. It is concluded that the overall risk for cross contaminations in biobanks is relatively low when following standard operating procedures (SOPs). We evaluated the potential sources in detail and summarised our results in a risk assessment spreadsheet which can be used for the quality management of biobanks.

**Key points:**

• *Identification of potential contaminants and their sources in LN storage tanks.*

• *Recommendations to reduce this risk of LN storage tank contamination.*

• *Development of a risk assessment spreadsheet to support quality management.*

**Supplementary Information:**

The online version contains supplementary material available at 10.1007/s00253-021-11531-4.

## Introduction

Biobanks are fundamental to future advancements in science, public health, and the bioeconomy. Their major role is the preservation and provision of biological resources for basic, industrial, agricultural, environmental and medical research and development, and for applications (OECD 2007). They collect, store (“bank”), and preserve reproductive organs, tissues, and cells of humans, animals, plants, and microorganisms. These valuable biological materials also enable the follow-up of scientific investigations, medical diagnostics, the development of forecast systems, biotechnological applications, as well as the conservation, assessment, and distribution of genetic resources of wildlife, food, and agriculture (Overmann 2015; Overmann and Smith 2017; Schüngel et al. 2014; Stock et al. 2018). State-of-the-art biobanks do not only provide access to high-quality biological materials but also to associated data, thereby enabling rapid response to disease outbreaks, like those of the Zika virus or COVID-19 (Peeling et al. 2020), or the Panama disease threatening banana production (García-Bastidas et al. 2019). They also facilitate progress in plant and animal breeding and protect (microbial) diversity required for human society. In the past decade, several biobanks have been successively joined to form large international research infrastructures which are capable of meeting complex challenges like the development of personalised medicine (Malsagova et al. 2020) including the Cancer Genome Atlas (Coppola et al. 2019).

The overall purpose of biobanks is to maintain the high quality, integrity, and functionality of the material. Therefore, samples are stored at ultra-low temperatures, usually in liquid nitrogen (LN) at − 196 °C or in the LN vapour phase (between − 140 and − 180 °C). Under these conditions, metabolic, physical, and chemical process rates are slowed down by more than ten orders of magnitude and hence do not affect the properties of the material even during long-term storage. To avoid ice crystal formation and osmotic stress during freezing, protocols are adapted differently for reproductive organs, viable tissues, cell types, and microorganisms by applying slow freezing or vitrification-based approaches. During slow freezing, samples are often treated with cryoprotectants such as dimethyl sulfoxide (DMSO) or glycerol cooled at a rate of approximately 1 °C min^−1^ to a temperature between − 50 and − 80 °C and subsequently transferred to LN. During vitrification, however, samples are treated by membrane-permeable cryoprotectants, e.g. ethylene glycol, glycerol, DMSO, or nonpermeable cryoprotectants, e.g. trehalose, sucrose, and then subjected to ultra-rapid cooling, which prevents ice crystal formation and converts the cell content into a so-called glassy state (Bojic et al. 2021; Panis et al. 2020; Sharma et al. 2017). For many microorganisms, freeze drying and liquid drying are often also successfully applied (Smith and Ryan 2012). If tissues are preserved for the purpose of DNA extraction, they are directly frozen in LN (Clarke 2009). The individual treatments during the whole pre-analytical workflow, including sample selection, sampling, sample transport, sample processing, and storage, affect the quality of the biological material (Malsagova et al. 2020). Especially, delayed specimen processing, variations in surgical manipulation, preservation condition, freeze–thaw cycles, or duration of storage (Zhou et al. 2015) can have a great impact on comparative studies and need to be optimised for translational research. To meet the growing requirements for translational research, harmonised standard operating procedures (SOPs), laboratory information management systems, and automation solutions have been developed over the last years (Coppola et al. 2019; Malsagova et al. 2020; OECD 2007, 2009). In Germany, the DIN EN ISO 20387:^2018 ^“Biotechnology–Biobanking–General Requirements for Biobanking” (DIN EN ISO 20387:^2018^) has been introduced to reach the highest possible quality levels by standardisation, harmonisation, and quality control (Baber and Kiehntopf 2019). As soon as these guidelines can be applied, high levels of transparency, comparability, and optimised cell viability and quality can be expected.

Additional potential risks affecting samples in biobanks might be a shortage in staff and infrastructure-related supply problems during pandemic situations (Parry-Jones et al. 2017), breakdowns or other disasters, and the cross contaminations of samples in cryotanks, particularly by microorganisms. To avoid the loss of material via staff shortage, supply problems, and disasters, spatially separated backup solutions are strongly recommended and often mandatory for biobanks. To overcome the risk of bacterial or viral cross contaminations, specific guidelines or risk assessments have not been developed yet. Although Schafer et al. (1976) already pointed out that LN storage tanks might be a source of laboratory infections, most previous reviews on biobanking cover only general information about biobanks (Coppola et al. 2019), laboratory operations (Cicek and Olson 2020), cryopreservation procedures (Bojic et al. 2021), or specific topics such as global health (Mendy et al. 2018) and reproduction medicine (Tao et al. 2020). So far, the potential of contamination in cryobanking was only discussed by Larman et al. (2014), Bielanski and Vajta (2009), Joaquim et al. (2017), and Vajta et al. (2015).

The present review provides an updated and comprehensive overview of the potential risks for microbial cross contamination in LN tanks in which organs, tissues, and cells of humans, animals, plants, and microorganisms are stored. Based on the available data, we describe potential contaminants and their sources in different types of LN storage tanks, evaluate the current awareness of 34 biobanks for cross contaminations, and provide possibilities to reduce this risk of contamination. This information was used to develop a novel risk assessment spreadsheet to support quality management of the biobanks by considering the possibility of cross contaminations which can now be included in standard operating procedures.

## The relevance of contaminations in storage containers

**Fig. 1:**
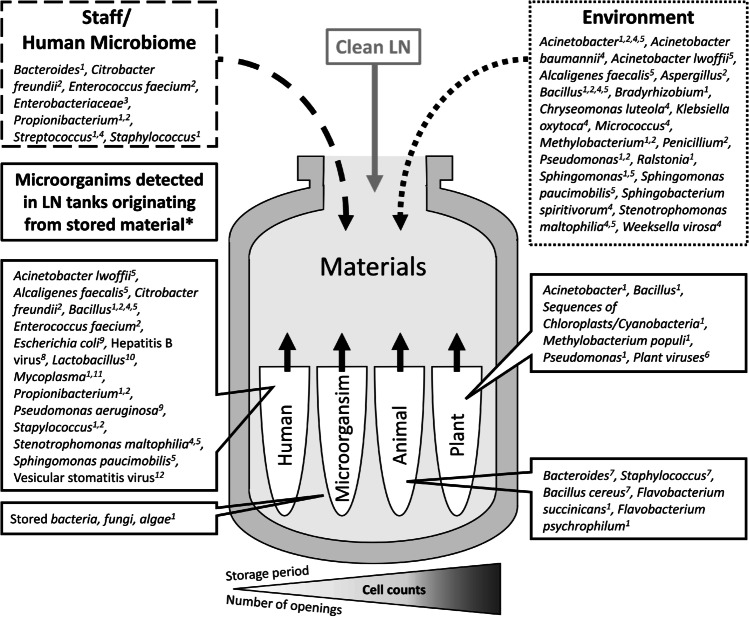
Overview on potential sources of microorganisms in liquid nitrogen tanks storing organs, tissues, and cells of human, animals, plants, and microorganisms. The shading of the arrow (cell counts) shows the increase in the number of microbial cells with increasing storage time and number of openings which indicates the likelihood of consequences for human health and welfare if the material is contaminated (light: low, dark: high). ^1^Bajerski et al. 2020, ^2^Fountain et al. 1997, ^3^Ramin et al. 2014, ^4^Morris 2005, ^5^Molina et al. 2016, ^6^Knierim et al. 2017, ^7^Pessoa et al. 2014, ^8^Tedder et al. 1995, ^9^Bielanski et al. 2003, ^10^Vitrenko et al. 2017, ^11^Drexler and Uphoff 2002, ^12^Schafer et al. 1976. *Microoragnisms detected in LN tanks most probably originating from the stored material itself displayed in boxes with solid lines

LN storage containers can be contaminated with microorganisms (Fig. 1). In 1995, a study reported that samples of bone marrow stored in LN were contaminated with hepatitis B virus after direct contact with tank detritus (Table 1). As a result, six multiply transfused patients developed icteric acute hepatitis B virus (HBV) infection (Tedder et al. 1995). Although the detailed route of transmission was not completely clarified, the case raised awareness that cross contaminations may occur in LN tanks. Theoretically, microbial contamination can be caused via transmission in the LN itself or via the contact of samples with contaminated outer surfaces, e.g. of containers or tanks (Fig. 1). Morris (2005) assessed whether ice sediments accumulating in LN storage tanks constitute a source of microbial contaminations. He found that sediments in different dewars from in vitro fertilisation clinics contained between 10^2^ and 10^5^ colony-forming units of bacteria per millilitre of melted sediment (the different bacterial species are listed in Fig. 1). In a subsequent, large-scale, systematic study, ice accumulating underneath the tank lids and along the rim, as well as debris at the tank bottom, were demonstrated to contain microorganisms in amounts detectable by culture-independent methods (up to 10^4^ cells per millilitre of ice (Bajerski et al. 2020). Thus, the formation of ice crystals that entrap microorganisms represents a major risk factor for contamination (Morris 2005; Schafer et al. 1976).

**Table 1:** Overview of the original research literature addressing contaminations in relation to liquid nitrogen (LN) storage

Take-home message	Contaminant	Stored/tested material	Study design	Literature
Low bacterial load of ice and sediment in storage tanks can be minimised by reducing ice formation and by using hermetically sealed sample containers	Technical environment (*Pseudomonas*, *Acinetobacter*, *Methylobacterium*), human microbiome (*Bacteroides*, *Streptococcus*, *Staphylococcus*), stored material (*Elizabethingia*, *Janthibacterium*)	LN/ice/debris phases of 10 biobanks storing different materials	Fluorescence microscopy and specific marker gene amplification	(Bajerski et al. 2020)
Contamination of storage containers and samples by possible transfer of “free” virus particles via LN	Virus	Embryo	Artificial experiment	(Bielanski et al. 2000)
Effective decontamination of dewars with sodium hypochlorite solution, the quaternary ammonium-based disinfectants and peracetic acid	*Pseudomonas aeruginosa*, *Escherichia coli*, *Staphylococus aureus*, bovine viral diarrhoea virus (BVDV), and bovine herpesvirus-1 (BHV-1)	Semen and embryos stored in dry shippers	Artificial experiment (LN contamination)	(Bielanski 2005)
Contamination of 45% of open devices vs. closed devices with no contamination after contact with artificial contaminated LN	*Pseudomonas aerigonosa*, *Escherichia coli*	Novel closed ultravitrification device (Ultravit)	Artificial experiment (LN contamination) and selective culturing	(Criado et al. 2011)
Low-level contamination of tanks and samples	Environmental and waterborne organisms	Peripheral blood progenitor cell and bone marrow	Selective culturing	(Fountain et al. 1997)
Potential risk for contamination of the vapour phase of LN demonstrating that such particles contained in liquid nitrogen are released back into the environment when nitrogen vapour cools down	Fungal spores and organic crystals, crop plant pathogens can be cotransferred	Programmable freezers and dry shippers (semen, embryos)	Artificial experiment (LN contamination)	(Grout and Morris 2009)
Cross contamination not probable, but microorganisms can survive LN storage	LN tanks: *Bacillus* spp., *Stenotrophomonas maltophila*, *Enterobacter* spp.; in sediments: *Acinetobacter lwoffii*, *Alcaligenes faecalis*, *Sphingomonas paucimobilis*)	Oocyte and embryo, open and closed vitrification	Selective culturing	(Molina et al. 2016)
Formation of ice crystals that entrap bacterial and fungal agents	General laboratory environment (*Klebsiella oxytoca*, *Acinetobacter baumannii*, *Weekseella virosa*, *Chrysenomonas luteola*), not from samples	IVF samples (human embryos and spermatozoa)	Selective culturing, scanning electron cryomicroscopy	(Morris et al. 2006)
Effective decontamination of vitrification carriers after three washes with certified ultraviolet sterile LN	*Pseudomonas aeriguinosa*, *Escherichia coli* and fungi (*Aspergillus niger*)	Human specimen	Artificial experiment (LN contamination) and selective culturing	(Parmegiani et al. 2012)
Effective decontamination of (sample) contaminated tanks, canisters, and sediment of companies and farms	*Bacillus cereus*, other bacteria and fungi	Animal semen	Culturing with swabs on selective media	(Pessoa et al. 2014)
No contamination of samples stored in closed vials, environmental microorganisms (also determined in the room air) in freezers and dry shippers; bacteria survive direct exposure to LN	Artificial: *Citrobacter freundii* and *Staphylococcus aureus,* environmental microorganisms	Embryos, spermatozoa of mutant mice	Molecular virus detection and swab cultivation of bacteria on selective media and artificial LN contamination	(Ramin et al. 2014)
Detection of infectious virus particles in LN tank caused by shattered glass ampoule	Infectious virus (vesicular stomatitis virus and others)	Viruses	Molecular virus detection	(Schafer et al. 1976)
Disease transmission after contact with contaminated LN caused by container leakage	Hepatitis B virus	Bone marrow samples	Molecular detection of DNA	(Tedder et al. 1995)
Effective removal of contaminated samples with washing and antibiotic treatment	Typical vaginal microbiota (e.g. *Lactobacillus*), soil/water inhabitants as known ventilation born/hospital contaminants	Foetal tissue	Combination of culture-dependent and PCR techniques	(Vitrenko et al. 2017)
Alternative cryoprotectant- and LN-free cryopreservation for spermatozoa with clean liquid air		Spermatozoa		(Wang et al. 2020)

The presence of the microorganisms observed in all phases of LN tanks can be explained by their (1) high abundance in the environment, (2) association with operating personnel, and (3) the introduction into LN tanks through the stored biomaterials (Fig. 1). A low abundance of environmental and skin microbiota was detected by culture-based (Fountain et al. 1997; Molina et al. 2016; Morris et al. 2006; Ramin et al. 2014) and molecular approaches (Bajerski et al. 2020) in the liquid and the vapour phase of LN tanks. In addition, contamination with fungi was observed in the sediments of some commercially operated tanks (Bielanski et al. 2003; Fountain et al. 1997; Pessoa et al. 2014). Fungal DNA in ice and debris occurred even in tanks storing material in the LN vapour phase, and even the implementation of air filtration systems did not prevent the occurrence of fungi (Bajerski et al. 2020). However, the main contaminant in tanks and tank sediments of animal breeding companies and farms was *Bacillus cereus* which originated from stored samples (Pessoa et al. 2014). Similarly, the fish pathogens *Flavobacterium succinicans* and *Flavobacterium psychrophilum* were exclusively found in samples from a LN tank in which fish samples were stored in glass flasks, and thus might originate from the cryopreserved material (Bajerski et al. 2020). The exceptional high amount of chloroplasts and a few bacterial taxa (*Elizabethkingia*, *Empedobacter*, and *Janthinobacterium*) in single tanks storing plants and microorganisms, respectively, likely is caused by material released from leaking containers (Bajerski et al. 2020).

In conclusion, each tank seems to have its own characteristic microbial community (Bajerski et al. 2020; Molina et al. 2016; Morris et al. 2006) and the potential for cross contaminations between samples may depend on a variety of factors that need to be evaluated separately.

## Biobank material and their possible contaminants

### Microorganisms

Microorganisms make up the main part of the earth’s biomass but only about 0.1% of all bacterial species have been isolated so far (Overmann 2013). Culture collections preserve and distributed pure cultures of microorganisms. Currently, 803 culture collections in 78 countries hold 3,293,173 strains of bacteria, fungi, viruses, and cell lines (http://www.wfcc.info/ccinfo) of which 511,630 of the strains from 141 collections are included in the global catalogue of microorganisms (http://gcm.wfcc.info/) (Wu et al. 2013). Some culture collections also offer associated services, training opportunities, and consultancy and, because of their much broader portfolio, are termed microbial domain Biological Resource Centres (mBRCs), (Overmann and Smith 2017). The database BacDive mobilises and integrates research data on a strain level from diverse sources providing standardised bacterial information as a digital biological resource (Reimer et al. 2019). The significance and future role of mBRCs have been highlighted in several previous reviews and book chapters (Janssens et al. 2010; Overmann 2015; Overmann and Smith 2017; Sharma et al. 2017; Flickinger and Smith 2009; Smith et al. 2013).

The isolation and characterisation of novel microbial strains are cost-intensive and the possibility to reisolate the same genotype from natural samples is highly unlikely (Overmann 2015). Thus, long-term preservation of microorganisms is of special importance and must ensure the morphological, physiological, and genetic stability of the conserved biodiversity. Long-term preservation can be performed by choosing ultra-low freezing (ultralow freezers), freeze drying (lyophilisation), or cryogenic freezing (cryogenic freezers) (Sharma et al. 2017). As microorganisms can survive severe conditions, i.e. in polar regions (Bajerski et al. 2017; Georlette et al. 2004), microorganisms are capable of surviving as such and without cryoprotectant in samples or freely in the tank atmosphere by reduction of metabolism and the formation of endospores (Georlette et al. 2004; Shimkets 2013). For example, *Citrobacter freundii* and *Staphylococcus aureus* survived the direct exposure to LN when subjected to LN on filter papers (Ramin et al. 2014), but even microorganisms that might grow at subzero temperatures down to − 15 °C (Mykytczuk et al. 2013) are not expected to actively metabolise in LN storage tanks. Usually, microorganisms are preserved by standardised cryopreservation protocols and with cryoprotectants such as DSMO or glycerol (Bajerski et al. 2018). Carbon-rich media such as Tryptic Soy Broth and supplemented sugars can also act as cryoprotectants themselves providing a favourable environment for the samples and their accompanying organisms (Vekeman et al. 2013). This can result in a higher cryopreservation success of the accompanying microorganisms compared to the actually stored target organism (Bielanski 1997). Furthermore, some microorganisms do not occur as a single isolate but as a mixture of organisms that cannot be (easily) separated, for example as phototrophic consortia (Overmann and Schubert 2002).

When different microorganisms are preserved in the same LN tank, each sample might act as a source of potential cross contaminant on its own, especially when containers that are non-hermetically sealed, such as screw cap tubes or fragile sample containers, are used.

### Plants

Storing plant material has been important since the beginning of agriculture, but the need to conserve numerous landraces became essential when they were replaced by a few modern varieties during the Green Revolution. Nowadays, plant genetic resources for food and agriculture (PGRFA) are preserved in 710 so-called ‘ex-situ genebanks’ storing about 5.4 million accessions from over 7000 genera. Most accessions are maintained as desiccation tolerant seeds under cold storage conditions, mostly at − 18 °C. However, accessions, which produce desiccation-sensitive seeds such as mango and coconut, or no seeds, i.e. garlic and cultivated banana, or those that require the maintenance of specific gene combinations such as potato, can only be maintained as vegetatively propagated plants, of which 13% are cryopreserved (WIEWS 2021). Recent overviews of current approaches are reviewed by Panis et al. (2020) and Wang et al. (2020).

Wild species are mainly conserved by over 370 botanical institutions, i.e. Millennium or the Australian Seed Bank Partnership, which collect and bank seeds of wild species (O’Donnell and Sharrock 2017). Comparable to PGRFA, > 20% of the plant taxa can only be maintained vegetatively and would require cryopreservation. Unfortunately, systemic documentation about cryopreserved wild species do not exist so far (Sharrock 2020). For both, for PGRFA and wild species, slow cooling or vitrification-based procedures (Uragami et al. 1989) are used to overcome the problems associated with ice crystal formation. Both procedures are successful for different species and the basis for the world’s cryocollections on apple, mulberry, elm and banana, potato, garlic, cassava, mint, and strawberry (Panis et al. 2020).

Healthy plant tissues are commonly colonised by bacteria or fungi which do not damage the host or elicit defence responses (Wilson 1995). These so-called endophytes vary in number and composition between plant species, genotypes, single clones, and even plant organs (Brader et al. 2017). In poplar hybrids (*Populus* spp.) grown in the field, about 53 bacterial taxa were found including *Proteobacteria*, *Actinobacteria*, *Firmicutes*, and *Bacteroidetes* (Ulrich et al. 2008). The majority of plant-associated bacteria derive from the soil environment, migrate to the rhizosphere and the rhizoplane of their hosts (Compant et al. 2010), and some proliferate also in subpopulations in in vitro plants (Quambusch et al. 2016). When in vitro plants are excised and shoot tips are exposed to the stressful conditions of cryopreservation, the process triggers massive proliferation and outbreaks of endophytes. The colonisation of these microbes emerges usually around the shoot tips during rewarming and compromises the ability of explants to regrow to a fully developed plant (Köpnick et al. 2018; Senula et al. 2018; Senula and Keller 2011). An increased number of subcultivations and suboptimal environmental conditions during in vitro plant growth alter the quality of the donor material and provoke endophyte growth after cryopreservation (Keller et al. 2011; Scherling et al. 2009). So far, plant-associated *Methylobacterium populi* could be detected by sequencing in LN tanks and might originate from plant material stored in non-hermetically sealed containers (Bajerski et al. 2020). During cryostorage, the endophytes must have already been present and able to distribute if the material was kept in the LN phase.

### Animal biomaterials

Biobanking of biomaterials from animals serves to improve livestock breeding, to decelerate natural losses in gene diversity, to prevent species extinction, and to recover endangered natural populations, such as in the case of the giant panda in China (Zhang et al. 2004) or the black-footed ferret in North America (Livieri 2011). Thereby, animal biorepositories help to understand the fundamental biology of unstudied species (Comizzoli and Wildt 2017), by preserving DNA, RNA, somatic cells, blood products, microorganisms, and healthy and diseased tissue samples. The storage of reproductive materials, i.e. viable semen for artificial insemination, and embryos for embryo transfer, and in vitro fertilisation support veterinary medicine and enhance the breeding, management, and propagation of endangered species (Groeneveld et al. 2016). However, due to a large number of different species, the research, propagation, and long-term storage of reproductive material is extraordinarily complex (Comizzoli 2015). While protocols from domestic and nonthreatened related species are typically applied to non-domestic and endangered species, reproductive technologies are usually species-specific (Prieto et al. 2014). It is estimated that reproductive biology is so far understood for 0.25% of the world’s 40,000 vertebrate species (Comizzoli 2015).

In addition, the success of biobanking differs between sexes. The male’s gametes are produced in large quantities and relatively easily accessible, and viable epididymal spermatozoa can even be obtained from dead or castrated animals. Therefore, sperm is often used for genome biobanking, and slow freezing protocols were widely developed for sperm of mammalian, fish, and avian species and is under development for other vertebrate species (amphibians and reptiles) as reviewed by Saragusty (2012) and Prieto et al. (2014). The cryopreservation of the gametes of females is challenged by the small number of cells, differences in developmental stages, and the invasive procedures needed to access the oocytes or embryos. Embryo cryopreservation has reached a commercial level only in the cattle industry (Saragusty 2012), whereas cryopreserved sperm is almost used entirely for artificial insemination (Wolkers and Oldenhof 2021). In general, only a few biobanks holding biomaterials from domesticated animals exist and are hosted by veterinary hospitals, zoological gardens, breeding and diagnostics companies, national farm animal genetic resource gene banks, research institutes, and universities (Groeneveld et al. 2016). For wildlife preservation, international collaborative projects between zoological gardens, aquariums, museums, and universities worldwide were initiated to create international databases and to coordinate the banking of DNA and cells from threatened animals. Examples are the Frozen Ark Project (www.frozenark.org/) launched in 2004 (Clarke 2009), CryoArks (https://www.cryoarks.org), the Amphibian Ark (www.amphibianark.org/), and the Biological Resource Bank of Southern Africa’s Wildlife (Bartels and Kotze 2006).

Animals are closely associated with microorganisms, mostly prokaryotes which colonise the gut and external surface of animals, as well as some reproductive organs (Eisthen and Theis 2016). Some microorganisms are highly specialised, e.g. 90% of the bacterial species in termite guts are not found elsewhere (Hongoh 2010). Evidently, preserving animal organs and tissues will also preserve the accompanying microbiota. However, the level of species-specific microbial load is not always known and may differ between populations, health status, age, season, and according to Hacquard et al. (2015) is influenced by intrinsic factors such as pH, oxygen level, nutritional availability, temperature, other microbes, and the host genotype.

### Human biomaterials

Biobanking of human biomaterials is mainly pursued for the purpose of reproduction/fertilisation or related to human diseases. Typical human-derived biospecimens such as blood, tissue, urine, or salvia run through a lifecycle from study design to an analysis by the collection, storage, and processing of the samples, and each sample has to be managed appropriately following harmonised SOPs according to the OECD definition for BRCs (OECD 2007; Vaught and Henderson 2011). The biobanking of human materials comprises, besides the infrastructure itself, all legal and ethical aspects, as well as the management of data associated with the stored biomaterials (Coppola et al. 2019; Malsagova et al. 2020). For reproduction, semen, eggs/oocytes, or embryos are preserved. Summarising conflicting opinions on open vs. closed vitrification systems, Argyle et al. (2016) concluded that the increased use of ultra-rapid cooling by vitrification in in vitro fertilisation (IVF) leads to better cryopreservation success than slow-freezing protocols and achieve pregnancy rates comparable to those with fresh oocytes. Another systematic literature comparison of open vs. closed vitrification in IVF did not show significant differences in cryosurvival or pregnancy between the two methods, and, due to the high heterogeneity of data, did not indicate clear advantages of closed vitrification procedures (Cai et al. 2018).

Microorganisms colonise all parts of the body as commensals or opportunistic pathogens. The total number of bacteria in the human body is estimated to be 10^13^, while the number of bacteria in the body is actually of the same order as the number of human cells, making up a total mass of about 0.2 kg (Sender et al. 2016). The individual human microbiome is highly personalised and metagenomic studies estimate that more than 1000 different species colonise the human body (Dekaboruah et al. 2020). The genera *Corynebacterium, Staphylococcus*, *Propionibacterium*, *Micrococcus*, *Malassezia*, *Brevibacterium*, *Dermobacter*, and *Actinobacter* are the main residents of the skin surface (Roth and James 1988), while *Streptococcus*, *Granulicatella*, *Gamella*, *Actinomyces*, *Corynebacterium*, *Rothia*, *Veillonella*, *Fusobacterium*, *Prevotella*, *Porphyromonas*, *Capnocytophaga*, *Neisseria*, *Haemophilus*, *Treponema*, *Eikenella*, *Leptotrichia*, *Lactobacterium*, *Peptostreptococcus*, *Staphylococcus*, *Eubacteria*, and *Propionibacterium* (Aas et al. 2005) as well as *Candida*, *Cladosporium*, *Saccharomycetales*, *Fusarium*, *Aspergillus*, and *Cryptococcus* occur in the human oral cavities (Wade 2013). Other microorganisms are associated with samples stored in the BRCs such as human biospecimens from the urinary system, gut, or respiratory tract (Dekaboruah et al. 2020). Therefore, the sample material itself and the whole process of sampling is usually not sterile (Bielanski and Vajta 2009; Ramin et al. 2014; Vitrenko et al. 2017); hence, cryopreserved human material cannot be considered “free” of microorganisms.

### Cell lines

Cell lines provide valuable in vitro model systems for medical research (Malsagova et al. 2020). Major cell line repositories are the American Type Culture Collection, the Leibniz Institute DSMZ-German Collection of Microorganisms and Cell Cultures GmbH, the European Collection of Authenticated Cell Cultures, the Japanese Cancer Research Resources Bank, RIKEN BioResource Research Center (Japan), and the Korean Cell Line Bank (Malsagova et al. 2020). Cell lines are characterised in depth to meet the highest quality standards. One focus is on microbial contaminations caused by bacteria, especially mycoplasmas, fungi, yeasts, and certain human pathogenic viruses (Uphoff and Drexler 2013; Uphoff et al. 2015). Bacteria of the genus *Mycoplasma* are parasitic bacteria in the class of Mollicutes that can cause infection in humans and other vertebrates (Drexler and Uphoff 2002). The genus *Mycoplasma* is a known contaminant during cell culturing which renders the biological resource useless due to the production of artefacts, such as altered cell metabolism, protein, RNA, or DNA levels (Drexler and Uphoff 2002). Consequently, the evaluation of experiments is impossible and, in the worst case, an important cell culture gets lost for future research and application. In a recent study, *Mycoplasma* was only detected on the molecular level at very low abundances in LN storage tanks (Bajerski et al. 2020). However, *Mycoplasma* contaminations need to be avoided and are achieved by the standardised screening of eukaryotic sample material before cryostorage.

## Routes of cross contamination in LN storage tanks

Aside from microbial contaminations of the samples themselves, there are additional routes for microorganisms to reach LN storage tanks and cross-contaminate samples (Fig. 1). The impact of LN, the surrounding tank atmosphere, and the sampling and handling are discussed below.

### LN as a source

It is often speculated that LN itself is a source of microbial contamination. Liquefied gases including LN are commonly manufactured under controlled and standardised conditions in so-called air separation units, which separate the dried and filtered atmospheric gases at very low temperatures (Bajerski et al. 2020; Molina et al. 2016). Validated analysis conducted by Air Liquide Medical GmbH (personal communication with Dr. Carsten Pilger) and Molina et al. (2016) could not detect any bacteria or fungi in LN which was directly derived from the manufacturing process. Therefore, we do consider freshly manufactured LN typically as free of microbial contaminants. Nevertheless, microbial contamination might occur during transport or transfer and some ubiquitous microorganisms can be expected to occur in the storage tanks (Bielanski and Vajta 2009). The gaseous and liquid nitrogen is the transfer media that are commonly shared by different samples and thus contaminated LN can become a source for contamination (Bielanski et al. 2000; Fountain et al. 1997). Overall, the microbial load of the LN phases is detectable but low (ice and sediment in cryotanks) or even below the detection limit (LN in cryotanks (Bajerski et al. 2020)).

### Storage system

The storage system has an important role in the distribution of microorganisms between the samples. Relevant factors are the type of storage (in LN or above LN in the vapour phase) and the use of open or closed storage devices. The risk of shattered glass ampules was investigated in 1978 which revealed that stored *Vesicular stomatitis* virus resulted in the contamination of LN with infectious virus particles (Schafer et al. 1976). Nowadays, most biobanks use LN-persistent screw-cap cryovials or straws.

Molina et al. (2016) evaluated cross contamination between aseptic closed or open straws stored in LN. No bacteria or fungi were found in any devitrification media or sample device (open or closed). However, bacteria (*Bacillus* spp., *Stenotrophomonas maltophila*, *Enterobacter* spp.) were found in all storage tanks tested and used for oocyte and embryo cryopreservation, indicating that microorganisms were present in LN and survived the storage (Molina et al. 2016). Interestingly, the exposure of 40 closed devices and 20 open devices to LN artificially loaded with *Pseudomonas aeruginosa* and *Escherichia coli* (Criado et al. 2011) showed that 45% of the open devices were contaminated whereas none of the closed devices showed colony growth. Similarly, after contact with LN loaded with three bovine viruses (bovine viral diarrhoea virus, bovine herpesvirus, and bovine immunodeficiency virus), 21% of the unsealed straws but none of the sealed devices were contaminated (Bielanski et al. 2000). Nevertheless, LN tanks and LN in the tanks should be regarded as potentially contaminated (Vajta et al. 2015) and open systems should be avoided to reduce the risk of contaminations.

### Sampling and tank environment

Aside from contaminants originating from stored samples themselves and transmitted via LN; microbes may have other origins and can enter the tanks during the handling by staff or from the surrounding atmosphere (Fig. 1). Sources of microbial contamination are skin-colonising organisms such as *Streptococcus*, *Propionibacterium*, and *Staphylococcus* which are introduced during sampling and handling of samples (Bajerski et al. 2020; Fountain et al. 1997). Furthermore, Fountain et al. (1997) investigated the risk of environmental contamination in LN storage tanks, which were used for both, storage in LN and in the LN vapour phase. The authors found low-level contamination with environmental and skin-colonising organisms in 4 of 5 storage tanks (*Bacillus*, *Corynebacterium*, *Staphylococcus*, *Streptocoocus*, *Aspergillus*, and *Penicillium*), the LN tank storing in the vapour phase was heavily contaminated with fungal elements (*Aspergillus* and *Penicillium* (Fountain et al. 1997)). Some environmental organisms were frequently found in cryotanks (*Acinetobacter*, *Bacillus* (Bajerski et al. 2020; Fountain et al. 1997; Molina et al. 2016; Morris 2005)), others (e.g. *Methylobacterium*, *Pseudomonas*, *Sphingomonas*, *Stenotrophomonas*) were detected in some of the named studies using culture-based and molecular approaches (Fig. 1). Ice crystals that form underneath the lids and on the rim of the tanks can especially entrap these microorganisms and have been identified as a potential contamination risk (Bajerski et al. 2020; Morris 2005; Schafer et al. 1976). Furthermore, fungal spores can be taken up by LN from the surrounding tank environment and released into tanks when nitrogen vapour cools down (Grout and Morris 2009).

In foetal tissues, the typical vaginal microbiota (e.g. *Lactobacillus* sp.) and airborne or hospital contaminants were detected, likely entering the samples during surgery and handling (Vitrenko et al. 2017). In animal biomaterials, additional contaminations must be considered when tissues and organs are not obtained under sterile conditions. Thereby, semen of most domestic species such as a stallion is typically collected for cryopreservation outside the breeding station (Wolkers and Oldenhof 2021). In addition, the collection method can have profound effects on the microbial load of sample materials. For example, retrograde flushing for the collection of ibex sperm appeared to reduce microbial contamination compared to the cutting method and resulted in a larger number of sperm cells surviving the freeze–thaw cycle (Santiago-Moreno et al. 2009).

The role of storage time, usage frequency, or sample load has been investigated and revealed no conclusive results. Studies with a smaller sampling size (40 samples of 5 tanks (Molina et al. 2016) or 10 samples of 3 tanks (Morris 2005)) showed no correlation between sampling load, microbial load, and usage time. Bajerski et al. (2020) reported an increase of bacteria with storage time and a number of openings analysing 89 samples of 27 tanks and demonstrated that the specific position of the storage container plays a role. In summary, to control the risk of microbial contaminations, the technical environment of the tank (air, water, filter, and supply systems) and the sampling and handling procedure has to be specifically evaluated to exclude potential contaminants from the tanks.

## Risk assessment and avoidance

### Survey on cryostorage and biobanking

**Fig. 2:**
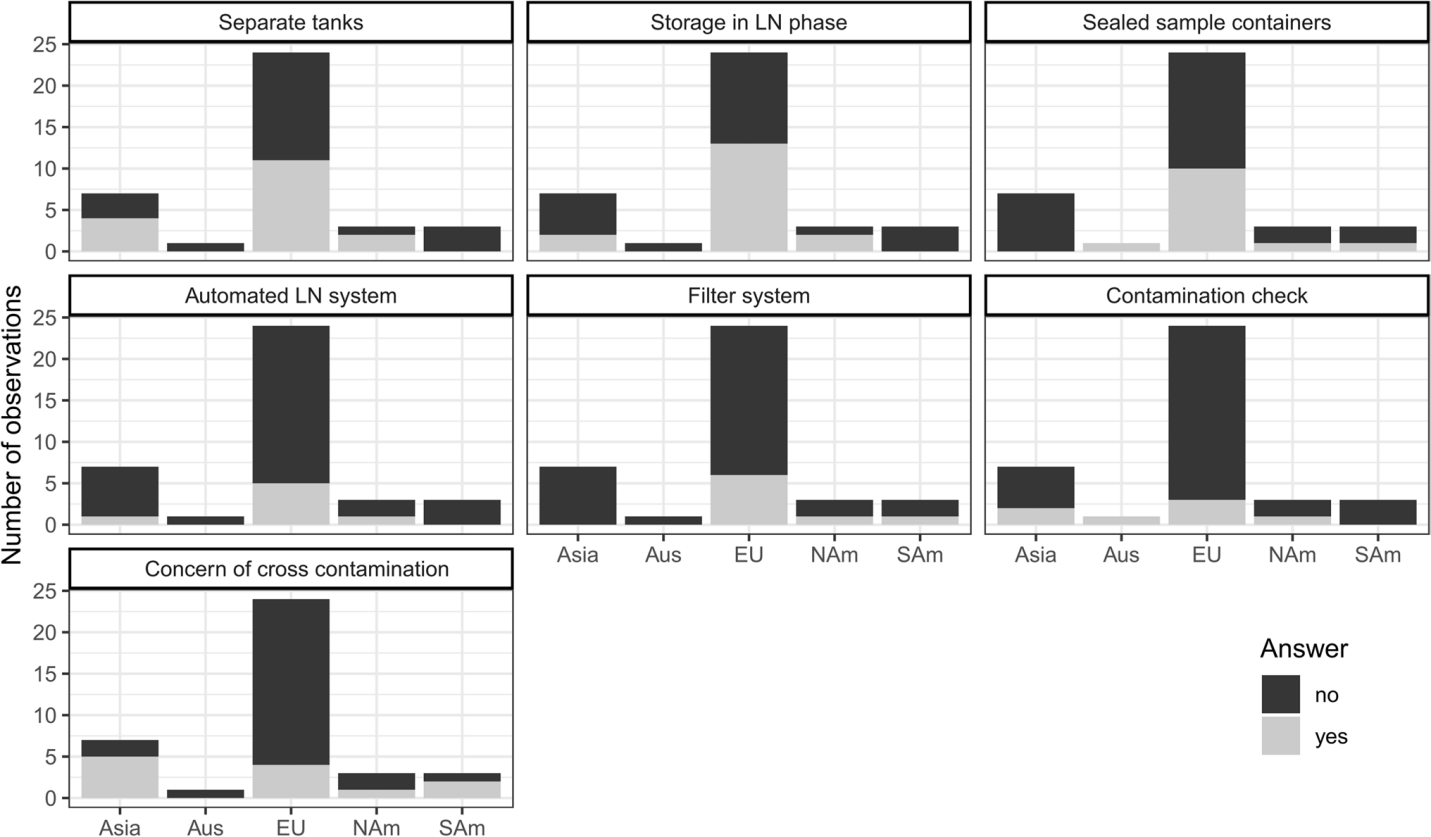
Results of a survey about common practice in cryobanks. In total, seven yes/no questions on cryostorage and biobanking were distributed using the scheduler provided by the German National Research and Education Network (DFN). The answers of 39 participants of 34 biobanks from 10 countries of 5 continents were evaluated using R version 3.6.2 (R Core Team 2019). LN, liquid nitrogen; Astr, Australia; Eurp, Europe; NrtA, North America; SthA, South America

To evaluate the potential awareness of cross contaminations in LN storage tanks, a survey was conducted between January 20 and April 6, 2021, by the authors (Table 2, Fig. 2). In total, seven yes/no questions on cryostorage and biobanking were distributed using the scheduler provided by the German National Research and Education Network (DFN). The answers of 39 participants of 34 biobanks from 10 countries of 5 continents were evaluated using R version 3.6.2 (R Core Team 2019).

**Table 2:** Results of a survey on cryostorage and biobanking**.** To evaluate the potential awareness of cross contaminations in liquid nitrogen (LN) storage tanks, a survey was conducted between January 20 and April 6, 2021 (Fig. 2). In total, seven yes/no questions on cryostorage and biobanking were distributed using the scheduler provided by the German National Research and Education Network (DFN). The answers of 39 participants of 34 biobanks from 10 countries of 5 continents were evaluated using R version 3.6.2 (R Core Team 2019)

Question	Answer	Counts	Relative frequency (%)
1. Do you use separate LN tanks to store different types of organisms, e.g. cell lines, microorganisms, and viruses?	No	22	58%
	Yes	17	45%
2. Do you store samples in the liquid phase of the LN storage tanks?	No	21	55%
	Yes	18	47%
3. Do you use sealed sample containers (e.g. straws)?	No	26	68%
	Yes	13	34%
4. Do you use an automated LN storage system?	No	32	84%
	Yes	7	18%
5. Do you use an air filter system in the LN storage room?	No	31	82%
	Yes	8	21%
6. Do you check for microbial contamination in LN tanks?	No	32	84%
	Yes	7	18%
7. Do you think that cross contamination of samples in LN tanks is of strong concern?	No	27	71%
	Yes	12	32%

About half of the participating biobanks stored different types of organisms, e.g. cell lines, microorganisms, and viruses in the same LN tanks (Table 2). Similarly, both LN phases (liquid and vapour) were used by ~ 50% of the biobanks. Some biobanks decided to move the majority of the stored material (e.g. cell lines, microbial culture, fungi, yeast, and bacteria) to − 150 °C freezers and only kept backup samples and some sensitive deposits in LN tanks.

Most of the participants store samples in sealed containers. Many biobanks have decades of experience using LN storage for sample preservation with a high percentage of viability and genetic stability and without observing any cross contamination. Although more than two-thirds of all participants do not assume that cross contaminations of samples in LN tanks are of strong concern, only 18% checked for microbial contamination in tanks and only about 20% used an air filter system. Up to this day, less than 20% of the participants use automated LN storage systems, but this may change with increasing sample numbers in the future as discussed below. In conclusion, although most participants consider the risk of cross contaminations in LN storage tanks as low, they prevent potential contaminations by using sealed devices and − 150°C freezers, indicating that the overall risk for cross contaminations is relatively low in biobanks employing SOPs.

### General recommendations

**Fig. 3:**
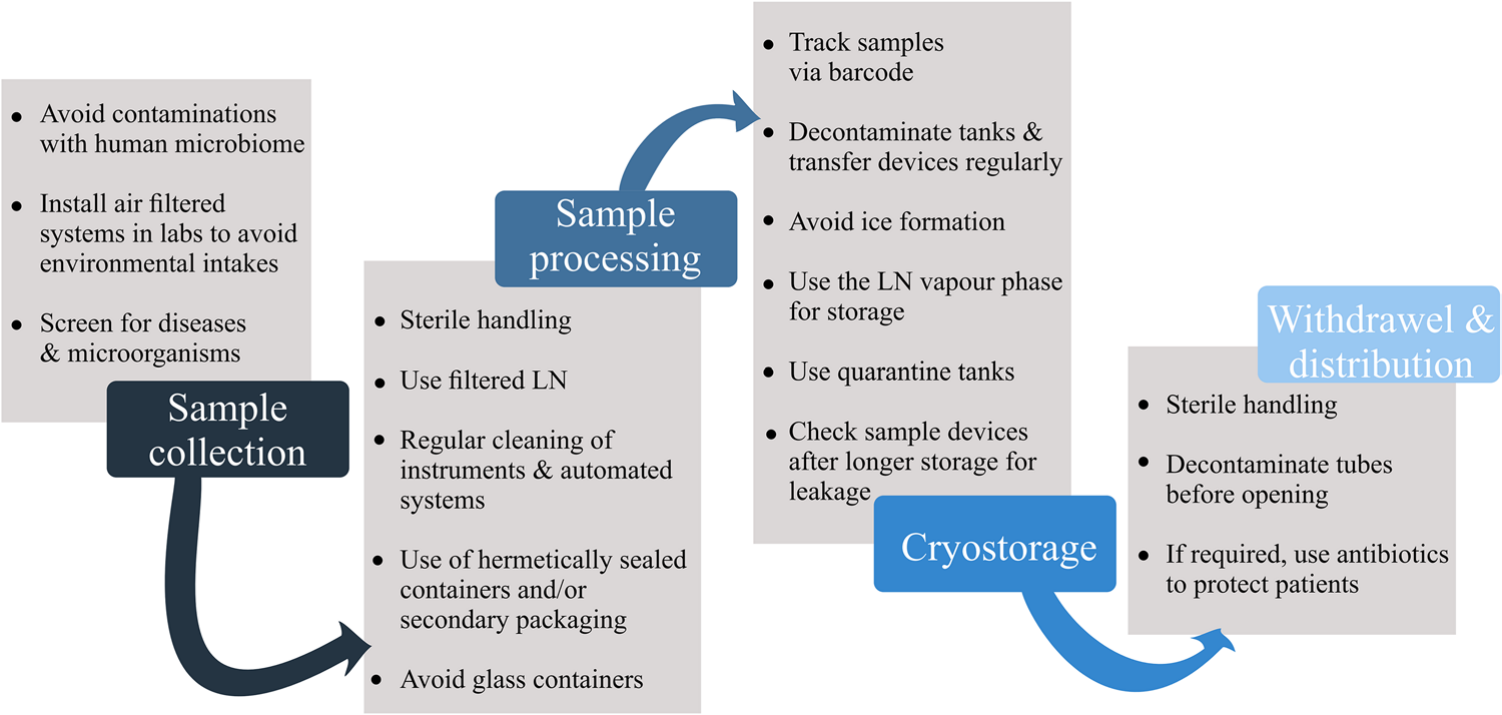
Recommendations to avoid microbial contaminations in liquid nitrogen tanks

Based on the results of the studies discussed, we propose several measures to reduce the risk for microbial contamination during sample processing and storage (Fig. 3).

1. Samples should be sampled and processed under sterile conditions to avoid contaminations with the human microbiome and environmental microorganisms.
2. Air-filtered systems in laboratories may reduce the frequency of environmental contaminants.
3. Samples should be cryopreserved contaminant-free if possible. Complex associations of organisms or communities need further research to meet their requirements for cryopreservation without posing a danger for cross contaminations of other samples. Therefore, we propose screening for diseases and specific microorganisms (*Mycoplasma* test, tests for axenic algae) and washing, if applicable, i.e. embryos can be washed prior to and after cryopreservation.
4. Factory-derived clean LN is often transported under non-sterile conditions (local distribution systems) and thus may not be regarded as sterile (Vajta et al. 2015). Therefore, the use of filtered LN during sample processing is recommended, especially for open vitrification approaches.
5. To maintain sterile conditions, regular cleaning of instruments including arms and cabinets of automated systems should be included to avoid the transfer of contaminants via outdoor surfaces of sample devices.
6. A potential risk for cross contaminations exists in open systems (Bielanski et al. 2000). Sample contamination can be avoided by using hermetically sealed devices and/or secondary packing (Bajerski et al. 2020; Bielanski et al. 2000).
7. To track potential cross contaminations, employ samples/containers equipped with a barcode.
8. Avoid ice formation and decontaminate LN storage tanks, sampling devices, and samples to minimize the risk for (cross-) contaminations (Bajerski et al. 2020). Dry (vapour) shipper and dewars contaminated with *Pseudomonas aeruginosa*, *Escherichia coli*, *Staphylococus aureus*, bovine viral diarrhoea virus, and bovine herpesvirus were successfully decontaminated using sodium hypochlorite, quaternary ammonium-based disinfectants, peracetic acid, or gas sterilisation with ethylene oxide (Bielanski 2005). Similarly, Pessoa et al. (2014) recommended decontaminating the tanks with 2% glutaraldehyde plus 70% ethanol. For vitrification carriers that are employed for IVF techniques, especially in non-hermitically sealed containers, three washes of vitrification carriers with certified ultraviolet sterile LN resulted in effective decontamination (Parmegiani et al. 2012).
9. To avoid a potential transmission by LN, storage in the LN vapour phase is recommended.
10. Overall, cross contamination of “clean material” with contaminated samples should be avoided by using “quarantine tanks”, if contamination is likely to occur or was detected before cryopreservation.
11. The manufacturers of sample devices guarantee the tightness only for a limited period of time. Therefore, sample devices should be checked for leakage after longer storage.
12. If samples were not sterile and/or are contaminated during surgery, sample handling, or washing, antibiotic treatment can remove contaminants in foetal tissues to protect patients from transplant-associated infections (Vitrenko et al. 2017).

### Risk assessment

To support the quality management of biobanks and maintain high sample quality, we propose a risk assessment that involves an analysis of the risks discussed in the previous paragraphs, an assessment of their impacts, and the implementation of controls and monitoring and reporting of the risk of microbial contaminations (Online Resource Table S1). To meet the quality management requirements of the biobanks, we developed a spreadsheet tool based on current standards such as ISO 31000:2009 (Standard B and Standard NZ 2009) that have been introduced to national standards, i.e. NIST Special Publication 800–30 for the USA (Ross 2012) or DIN ISO 31000:2018 for Germany (DIN 2018). In accordance with the previous overview, the threat of microbial contaminations can be divided into the potential risk based on their origin, namely contaminations via (1) the samples themselves, (2) LN, (3) human microbiome, (4) the environment, and (5) cross contaminations. To evaluate the impact of each specific risk, the likelihood (L) and impact (I) of the thread are evaluated based on semiquantitative values between 1 (less likely) and 10 (very likely), and a reciprocal approach 1 (very likely) and 10 (less likely) applies to the probability of detection (D). Since the consequences of microbial contamination depend on the sample material and the final usage, we distinguished between human (H) and animal (A) tissues for reproduction, transplantation and screening; plant material (P) and microorganisms (M) for maintenance and reproduction, and human and animal tissues for DNA extraction (HAD). Overall, the risk level for contaminations is low (risk level 1 to 30 of 100). We assume that contaminations are easily detectable by molecular approaches (detection probability 1). Microbes occur less likely in LN and have the lowest impact (risk level 1). Contaminations with environmental microorganisms and the human microbiome might yield comparable results but affect the samples differently (risk levels 2 to 10). Microorganisms in the samples and cross-contaminations in the LN storage are more likely, show higher impacts and are estimated between risk levels 2 and 30, especially the impact on human and animal tissue used for reproduction and transplantation is higher compared to material only stored for DNA extraction.

## Conclusions and future challenges

In the present overview, we have shown that microbial contaminations have been detected in LN storage tanks by culture-based and molecular approaches and originated from the samples themselves, LN, the human microbiome, environment, and can cause cross contaminations via LN transmission. The evaluation of the sources by the risk assessment spreadsheet tool (Table 2) indicated that LN is most likely not the source of serious contaminations. However, the samples themselves and the exchange of contaminants between samples in the LN storage tanks have the highest probability and at the same time the strongest impact for microbial contaminations. Some contaminants may not be considered risky because they coexist with specific organisms and are cryopreserved together. In any case, our survey showed that awareness exists for microbial contaminations of containers in cryocollections. Most participants prevent potential contaminations by using sealed devices and − 150 °C freezers. There are case studies that showed serious problems after contamination of samples and it has been revealed that in some organisms, the presence of microbial contamination can influence the recovery of reproductive organs for human, animal, and plant tissues after cryopreservation and transplantation. Therefore, a greater awareness of potential transmission routes is needed. Furthermore, we recommend the establishment of air filter systems, sterile conditions throughout the sample processing, the contaminant screening, and the regular decontamination of the LN storage tanks.

A future challenge will be the increasing number of biomonitoring, clinical studies, searches for diagnostic markers; the successes in fertility/viability preservation, and the conservation of wild animal species and plants, which together will lead to a greater demand for high-performance BRCs that run cryostorage facilities. The larger number of samples and the number of large-scale repositories will increase the risk of contaminations especially when different sample types are mixed and tank size increases. To optimise the handling of a large number of samples, laboratory information systems (LIMS) including barcode tracking, the adoption of harmonisation protocols such as DIN EN ISO 20387:^2018^ (Furuta et al. 2018), and large-scale, fully automated storage systems need to be implemented. However, as biobanks are the basis for future medicine, research, and genetic resources conservation, more original research is necessary to further elucidate potential risks and problems accompanying biobanking.

## Supplementary Information

Below is the link to the electronic supplementary material.

Supplementary file1 (XLSX 17 KB)
